# Fractal, diffraction-encoded space-division multiplexing for FSO with misalignment-robust, roaming transceivers

**DOI:** 10.1038/s41598-022-06660-3

**Published:** 2022-02-17

**Authors:** Xiaojing Weng, Luat T. Vuong

**Affiliations:** Department of Mechanical Engineering, University of California at Riverside, Riverside, 92521 CA USA

**Keywords:** Fibre optics and optical communications, Imaging and sensing, Optics and photonics, Electrical and electronic engineering

## Abstract

For free space optical (FSO) communication, a small misalignment of the transceivers may result in link failure or severe performance degradation. It can be difficult to track the narrow optical beams over long distances. Here, we propose “diffractal space-division multiplexing” (DSDM), an FSO transmission system capable of supporting misaligned roaming transceivers. This system enables spatial multiplexing for enhanced data capacity with partial off-axis beam reception. We numerically simulate and analyze the performance of the DSDM system with a particular focus on the divergence angle, roaming area, kernel bit-error-rate (K-BER), and fractal order. Our simulation results achieve K-BERs of 10$^{-3}$ with $81\times 81$-pixel fractal beams at link distances of 2.5 km when the receiver sizes are 30$\%$ of the effective beam diameter.

## Introduction

In the past, in order to achieve higher data transmission capacity, communication networks have embraced different modulation and multiplexing schemes^1,2^. Commonly used multiplexing techniques in optical fiber communication today include space-division multiplexing (SDM) or spatial multiplexing, wavelength-division multiplexing (WDM) using disjoint frequency bins, orthogonal frequency division multiplexing (OFDM) or coherent WDM (CoWDM) using spectrally overlapping yet orthogonal subcarriers, and polarization-division multiplexing (PDM) using both orthogonal polarizations supported by a single-mode fiber for independent bit streams^1,3^. In real applications, multiplexing techniques are combined to further increase the channel capacity^4–6^. Among these approaches, SDM has recently drawn sufficient attention as the space dimension is still not fully developed^7,8^, particularly with free-space optical (FSO) communication systems.

One potential approach for SDM uses beams with orbital angular momentum (OAM)^9–12^. Since OAM states are mutually orthogonal, they are simultaneously transmitted or multiplexed along the same beam axis and demultiplexed at the receiver. For the same carrier frequency, the system’s *aggregate capacity* is equal to the number of system state modes. Indeed, OAM-multiplexed systems have reported Tbit/s-scale transmission rates over free space^10^. Since then, Wang et al. have experimentally demonstrated free-space data links with aggregate transmission capacity of 1.036 Pbit/s and a high spectral efficiency of 112.6 bit/s/Hz using 26 OAM modes simultaneously with other multiplexing technologies^13^. However, since multiple OAM states are multiplexed along the same beam axis, coaxial propagation and reception are required, which means that coherent, OAM-multiplexed links are sensitive to misalignment^14,15^. For FSO SDM systems at distances beyond thousands of meters, this alignment is a critical challenge when transmitters and receivers are not fixed^16^.

Another approach to spatial multiplexing is multiple-input multiple-output (MIMO), in which multiple independent bit streams are transmitted simultaneously and multiple aperture elements are employed at the transmitter/receiver. As a well-established technique in radio wireless systems^17,18^, MIMO could provide capacity gains relative to single aperture systems and increase link robustness for FSO communications^19^. In practice, MIMO is prone to interference between the transmitted beams at different aperture elements. This interference arises when these apertures are not sufficiently spatially separated^20–22^.

**Figure 1:**
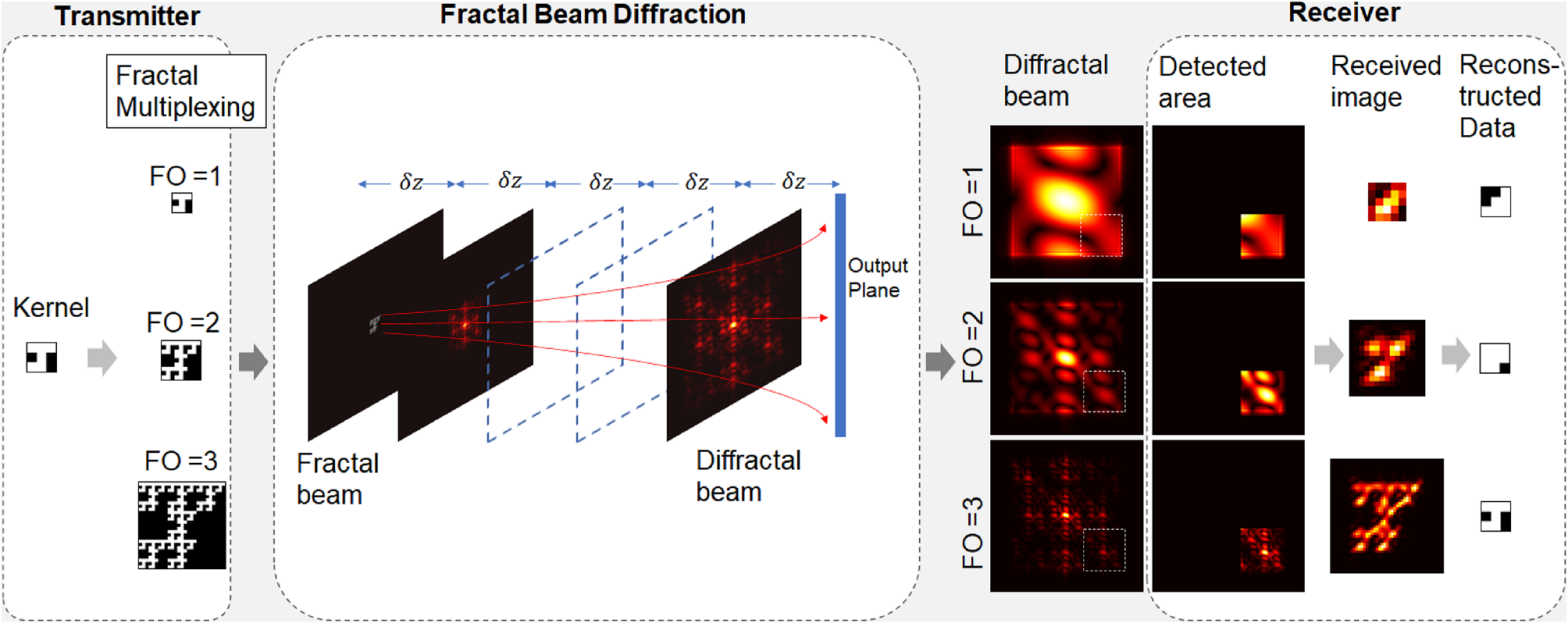
The concept of diffractal space-division-multiplexing (DSDM) in optical wireless communication. This schematic illustrates the data kernel pattern “J” and transmitted patterns with fractal order FO = 1,2,3. The transmitted pattern diffracts as it propagates to the receiver. Only a partial off-axis portion of the far-field beam is captured and reconstructed. The FO = 3 data is reconstructed accurately.

In this paper, we demonstrate the novel paradigm of diffractal space-division multiplexing (DSDM). Diffractals are the waves that have encountered fractals, which are visually-complex iterations of simple patterns^23–26^. Fractal geometries and diffractal scattering have attracted widespread attention in many branches of science and engineering such as digital image processing, especially image compression^27,28^ and antenna design^29–33^. Such applications exploit a high level of information redundancy and sparsity, which stems from the self-similar geometry of fractals or diffractals.

The unique property of diffractal redundancy enables misaligned or non-coaxial roaming transceiver^34,35^. The proposed DSDM system involves: multiplexing (where the transmitted data is a fractal from kernel data), diffraction encoding (when the beam propagates to the receiver), and demultiplexing (which is a hybrid system, composed of an optical receiver and a software threshold algorithm) (Fig. 1). The most closely related work to DSDM in FSO is by Moocarme et al., where diffractal multiplexing is demonstrated with a 4-F lens system using on/off keying (OOK)^34^. In the focal plane or far field, a portion of the Fourier transform of the transmitted fractal pattern contains sufficient information to recreate the entire original (sparse) signal^36,37^. In our study, DSDM employs an intensity modulation/direct detection (IM/DD) scheme, which is common for FSO communication systems^38^. Other research has begun to explore the potential of non-IM/DD or non-OOK fractal representations^39^.

DSDM offers several important advantages:

- Sparse sampling: Due to the self-similar geometry, the diffraction patterns of fractals contain redundant information. Thus, receivers that sample a portion of diffractal beam collect sufficient information for kernel reconstruction.
- Robust to misalignment: DSDM enables a roaming area for the non-coaxial receiver.
- High transmission capacity: DSDM could transmit multiple bits simultaneously using a single transmitter/receiver aperture pair. Additionally, DSDM may be used in practical free-space propagation systems to achieve higher transmission capacity in combination with other degrees of freedom, such as polarization and wavelength multiplexing.

One reason why DSDM may be underexplored is due to diffraction issues (i.e., diffractals generate a wide cone of high spatial frequencies as they propagate, which is counter to current paradigms for FSO). Additionally, the strong diffraction from irregularly corrugated beams is a challenge to simulate reliably: most theoretical work related to diffractals focuses on 1-D propagation or the far-field Fourier transform of a fractal. To our knowledge, there is no current work that examines the intermediate process of diffraction-encoding and mid-field propagation of diffractals explicitly for FSO, as we provide here. In order to advance our understanding of the novel approach with diffraction-encoding, here we establish basic parameters of the fractal propagation to the receiver: we measure the reconstruction accuracy and robustness of DSDM.

## Methods

### Description of the numerical simulations

We implement the angular spectrum split step method for free space propagation. The split-step propagation method is a popular approach for modeling electromagnetic wave propagation through the atmosphere by numerically solving the parabolic wave equation^40^. The optical field $U( x,y,z_0+\delta z )$ using the angular spectrum method is given by:

1 $$\begin{aligned} U( x,y,z_0+\delta z ) \,\,=\,\,\exp \left[ ik\int _0^{\delta z}{n_1\left( x,y,z \right) dz} \right] \times {\mathscr {F}}^{-1}\left[ \exp \left( i\delta z\sqrt{k^2-4\pi ^2( f_{x}^{2}+f_{y}^{2} )} \right) {\mathscr {F}}\left[ U( x,y,z_0 ) \right] \right] \end{aligned}$$

where ${\mathscr {F}}$ is the operator of Fourier transform. Thus, the numerical propagation of the field through the atmosphere is reduced to two independent steps:

1. Propagation through free space to a distance $\delta z$ using the Fourier-space operator:
  2 $$\begin{aligned} h(x,y,z) = \exp \left( i\delta z\sqrt{k^2-4\pi ^2( f_{x}^{2}+f_{y}^{2} )} \right) \end{aligned}$$
2. Multiplying the resultant field by a phase function that represents the effect of the refractive index fluctuations of the medium over the same distance :
  3 $$\begin{aligned} t( x,y ) \,\,=\,\,\exp \left[ i\theta ( x,y ) \right] \,\,=\,\,\exp \left[ ik\int _0^{\delta z}{n_1( x,y,z ) dz} \right] \end{aligned}$$

In this paper, we assume that the fractal beam propagates in a vacuum or $n_1( x,y,z )=1$ and *t*(*x*, *y*) is a constant. In other words, the diffraction is calculated in a single numerical step. An absorbing boundary is used to ensure that the power is conserved within the computational window or the result is repeatable with wider grid widths.

### Fractal beams

The kernel represents the information in the transmitted beam and is a binary $s \times s$ matrix, where $s=3$ in this paper. The fractal beam is generated with repeated Kronecker products of the kernel with itself, which is $U\left( x,y,z=0 \right) =K\otimes K\otimes \cdots \otimes K$, where *K* is the kernel matrix. When the beam’s fractal order (FO) is equal to *n*, the Kronecker product repeats *n* times. Therefore, the matrix size of each transmitted fractal beam is $s^n \times s^n = s^{2n}$. One element of the matrix is defined as one pixel when plotting the matrix as a image. Similarly, each transmitted fractal contains $s^{2n}$ pixels. Table 1 shows fractal beams used in this paper with different kernels and FOs. Some of the fractals generated by this process are well known: for example, in this paper, the iterated substitution of a $3\times 3$ kernel matrix of ones with removal of the center element, named kernel “O”, is referred to as a Sierpinski carpet.

**Table 1:**
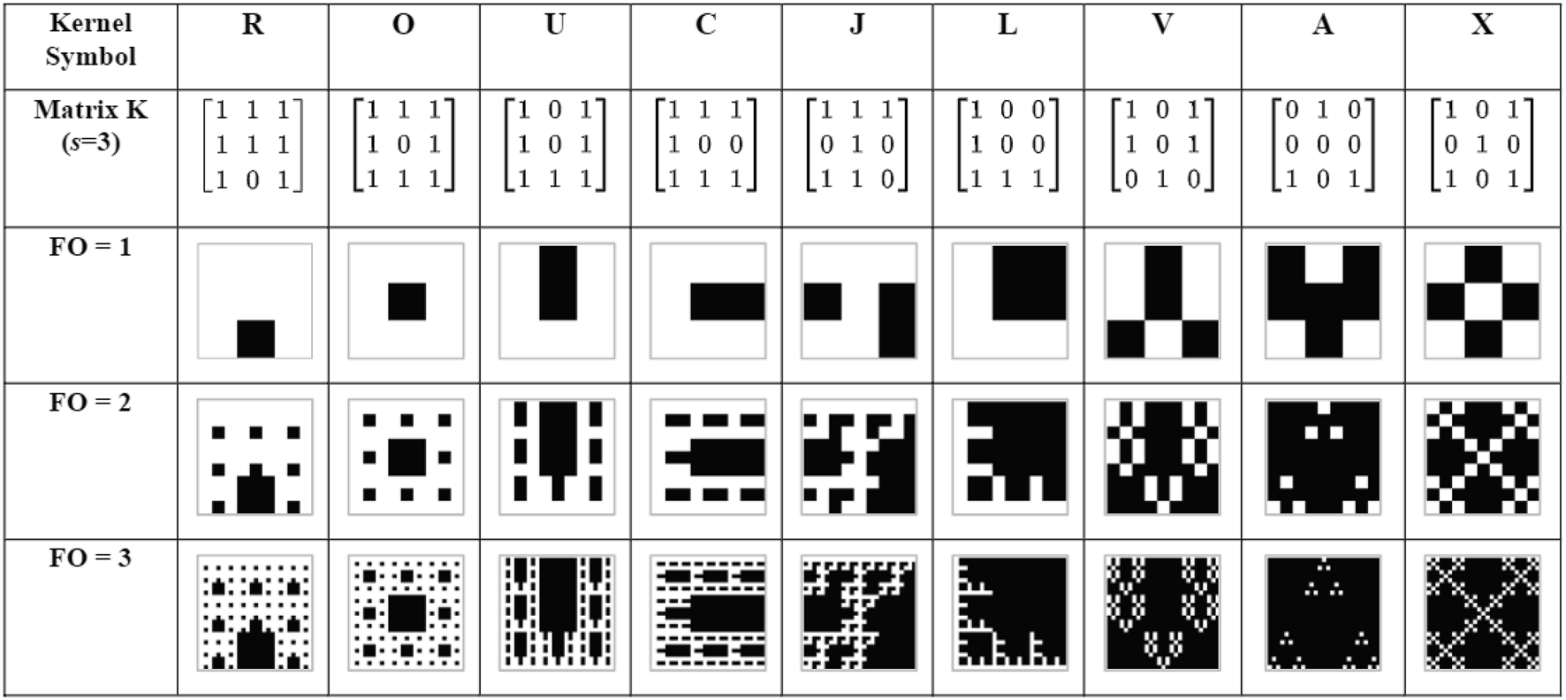
Fractals of some kernels with FO = 1, 2,3.

### Detection and reconstruction

DSDM detection and reconstruction involves both optics and software. The optics of the receiver consist of a convex lens and a CCD camera. The camera is located at the focal plane of the lens and captures the intensity pattern of the Fourier-transform of the detected beam. This received camera image is similar to the initial data kernel.

**Figure 2:**
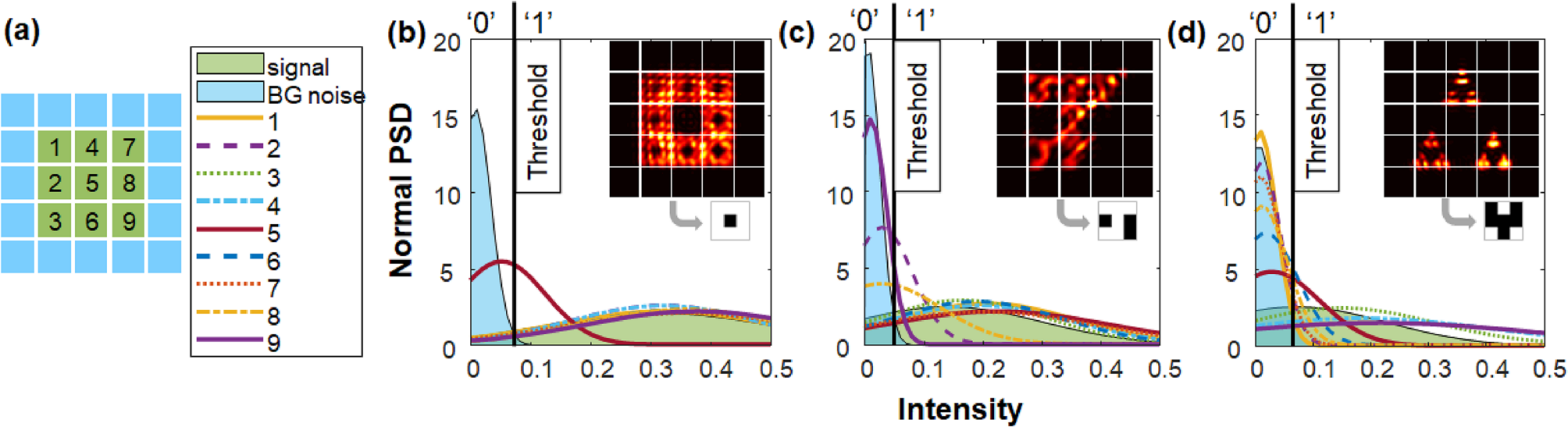
The thresholding algorithm. (**a**) Channels 1–9 are separated sub-blocks of the central signal (green areas) from up to down, left to right. Blue areas are background noise. (**b**–**d**) illustrate the reconstruction of kernels “O”, “J”, and “A” respectively. Each figure shows the normal curves of the signal (shaded green), background (shaded blue), and channels 1–9 sub-blocks. The threshold is the cross point of the normal curves of the signal and background noise. Peak points of channels 1–9 are then compared to the threshold to decide weather they are ‘0’s or ‘1’s.

On the software side, a thresholding algorithm is designed to reconstruct the kernel from the intensity profile of the received image. The threshold value is the crossing point of two normal distribution curves of active signal and background areas (green and blue areas in Fig. 2a, respectively). Mean $\mu$ and standard deviation $\sigma$ calculated from the signal and background area are used to plot these two curves of the normal point spread function (PSF) with the equation $y\,\,=\,\,\frac{1}{\sigma \sqrt{2\pi }}e^{-\frac{\left( x-\mu \right) ^2}{2\sigma ^2}}$ (see the normal curves with blue and green shade in Fig. 2b–d). After determining the threshold, the central active signal area of the received image is separated into nine sub-blocks and each sub-block corresponds to one binary bit of the reconstructed data. The mean value $\mu$ of each sub-block is compared to the threshold to decide whether this bit is ‘1’ or ‘0’. In Fig. 2b–d, channels 1–9 are nine normal curves corresponding to these nine sub-blocks. If the curve’s peak point is at the left side of its threshold, the reconstructed bit is ‘0’, otherwise is ‘1’. For example, as shown in Fig. 2b, because the peak point of the channel 5 curve is the only one at the left side of its threshold, the fifth bit of reconstructed data is ‘0’ and all the other bits are ‘1’s, which correctly reconstructs the kernel “O”. For the kernel “J”, the peak points of channel 2,8,9 curves are at the left side of its threshold (Fig. 2c). Thus, the second, eighth and ninth bits of the reconstructed data are ‘0’s. Similarly, for kernel “A” (Fig. 2d), the third, fourth and ninth bits are ‘1’s because their corresponding peak points are at the right side of its threshold.

## Results

### Diffractal beam divergence

Diffractal beam divergence is an important design consideration because it defines the effective roaming area for the receivers. In other words, it defines how far the receiver could travel from the center axis of the transmitted beam. From diffraction theory, a spatially corrugated beam such as a fractal diverges faster than a Gaussian-profiled beam. We numerically propagate diffractals with kernels as large as $3\times 3$. The total transmitted power is unit normalized and the total pixel number varies with FOs and kernel shapes so that the power contained in one transmitted pixel varies case by case. The pixel size ($W_{px}$ = 2 mm) and wavelength ($\lambda =1550$ nm) are used in this paper.

**Figure 3:**
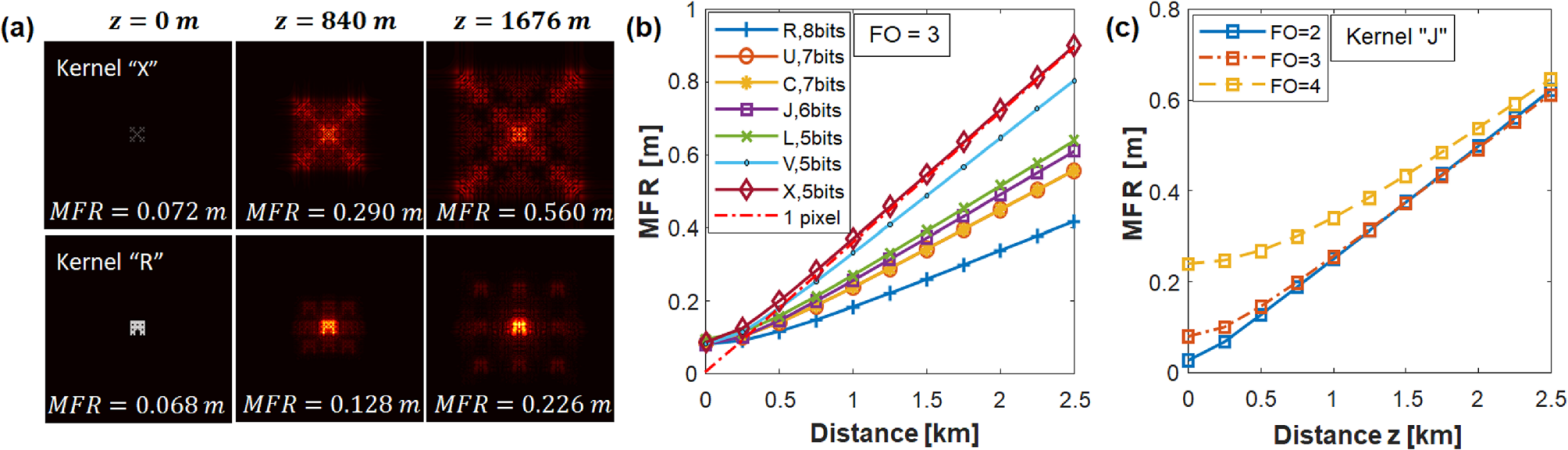
(**a**) Propagated beam profile of electric-field intensity at *z* = 0, 840 and 1676 m for kernel “X” and “R”. (**b**, **c**) MFR as a function of propagation distance *z* for (**b**) different data kernels with fixed fractal order FO = 3 and (**c**) different fractal orders FO = 2, 3, and 4 of the kernel “J”.

For the calculation of diffracted beam radius, the concept of beam mode field radius (MFR) is employed and given by the equation:

4 $$\begin{aligned} MFR\,\,=\,\,\sqrt{\frac{\iint _{-\infty }^{\infty }{\left| u\left( x,y \right) \right| ^2\left( x^2+y^2 \right) dxdy}}{\iint _{-\infty }^{\infty }{\left| u\left( x,y \right) \right| ^2dxdy}}} \end{aligned}$$

where *x* and *y* are the transverse spatial coordinates and *u*(*x*, *y*) is the electric field of the diffractal beam. The fraction of the total beam power within the maximum roaming area is less than or equal to $1-1/e^2$. Different kernel shapes have different degrees of diffraction. As illustrated in Fig. 3a, the kernel “R” diffracts at half the rate as the kernel “X”. The upper limit for the beam divergence speed is that for a single pixel. A diffractal with the kernel “X” spreads almost as much as a single pixel at the same propagation distance. We observe that the speed of the divergence scales approximately with the number of internal edges in the kernel shape or with the largest independent block length of the kernel.

In fact, even the “slowest” diffractals diverge at a rate much greater than a Gaussian beam of the same initial waist. For example, the kernel “R” diverges at a rate 26 times faster than a Gaussian beam with the same waist radius. As shown in Fig. 3b, the initial beam radius of the kernel “R” is around 10 cm. This beam radius increases by a factor of $\sqrt{2}$ after a propagation distance of *z* = 0.8 km. Meanwhile, the Rayleigh length of a Gaussian beam with a 10-cm waist radius is 21 km, given by^41^ $z_{R}=\pi /\lambda (w_{0})^2$, where $w_{0}$ is the beam waist. Compared to Gaussian beams, this extreme divergence of diffractals results from their intrinsic property of high geometric complexity. However, many aspects of diffractals at long distances are similar to Gaussian beams. For example, the diffractal beam radius MFR increases linearly with distance in the far field. This convergence, which depends on the kernel shape but not on FO is illustrated in Fig. 3c. With the same kernel shape and pixel size, beams with different FOs have different initial beam waists but similar far-field MFRs.

The diffraction pattern changes with the propagation distance *z* and this process of diffraction provides part of the signal spatial encoding. Therefore, the distance between transmitter and receiver influences the system’s reconstruction accuracy. We use the concept of a “Fraunhofer distance” to quantify this diffraction distance of the “far-field” region:

5 $$\begin{aligned} z_{DFF} > 2 s^{2n} L_{dfpx}, \end{aligned}$$

where *n* is the fractal order and $L_{dfpx} = \pi /\lambda (W_{px}/2)^2$ is the confocal parameter for a single pixel of width $W_{px}$.

Our simulations suggest that, in order to fully take advantage of the diffractal encoding, the receiver should be at a distance $z>z_{DFF}$ from the transmitter, where the diffraction pattern doesn’t change shape with distance. If $z<z_{DFF}$, the diffraction pattern differs significantly from that observed at infinity and varies with distance. We refer to this condition as “partial diffraction encoding” in this paper. Although the DSDM performance at $z<z_{DFF}$ is not optimal because of partial diffraction encoding, accurate image reconstruction is still achieved when the receiver aperture is sufficiently large.

### Influence of the roaming area

In the sections below, we calculate the kernel bit-error-rate (K-BER) to evaluate image reconstruction accuracy and DSDM system performance. The radius of the maximum roaming area for the receiver is equal to $\sqrt{2}$MFR. To draw statistics, we move the receiver over 4000 random locations across the diffractal beam (within maximum roaming area) to calculate the K-BER. The receiver aperture can be significantly narrower than the beam MFR and misaligned to capture light off-axis. The K-BER is calculated based on a fixed kernel rather than random kernels and provides a measure for kernel image reconstruction accuracy.

**Figure 4:**
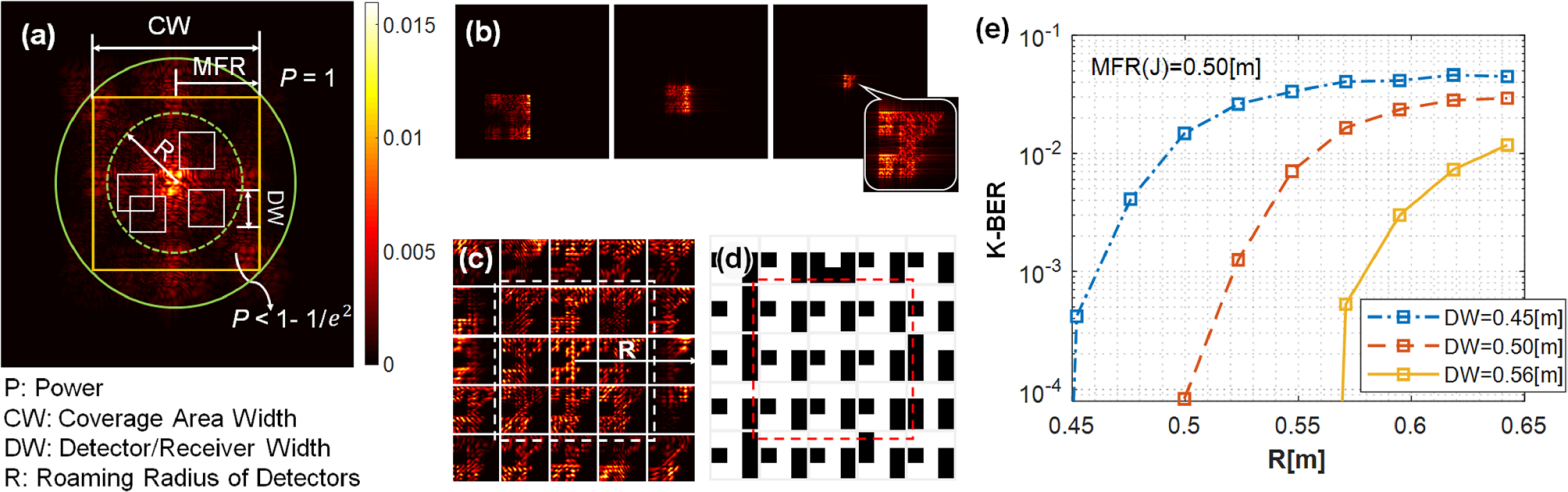
(**a**) The far-field beam pattern for the kernel “J” and definitions of coverage area (CW), receiver width (DW), and roaming radius (R). Several randomly-positioned receivers are shown (white squares). (**b**) From left to right, optical deconvolution of an off-axis receiver sized at DW = 0.75 m. The captured area represents 5.8% of total beam power and 16% of the coverage area. **c** Deconvolved images and **d** reconstructed data over 25 equally-spaced patches across the coverage area. The dashed-line squares indicate a possible roaming area wherein the sampled, reconstructed 9-bit images are all correct. **e** K-BER versus roaming radius (*R*) for different receiver widths (DW). The transmitted kernel is “J” with FO = 4 at *z* = 2.5 km.

Longer propagation distances result in larger roaming areas. The receiver captures only a portion of this diffractal beam. Figure 4a shows the definitions of receiver detector width (DW) and roaming radius (R) with respect to the beam MFR. The green dashed-line circle represents the possible roaming area (R $< \sqrt{2}$MFR) and the green solid-line circle is defined as the maximum roaming area (R = $\sqrt{2}$MFR).

As expected, the error probability increases as the receiver moves away from the far-field beam center axis and the error probability decreases with larger receiver areas. The roaming radius of the receiver directly influences the K-BER performance. Figure 4e illustrates the reconstructed K-BER as a function of the roaming radius *R* at a propagation distance of *z* = 2.5 km without turbulence, where the kernel is “J” and FO = 4. Figure 4b depicts the deconvolution process of a misaligned receiver at an arbitrary location. Figure 4c shows received images at locations shifting equally from left to right, up and down within the coverage area. Figure 4d shows the corresponding reconstructed data for these received images. In general, as the receiver moves farther away from the center of the diffracted beam, the K-BER gradually increases; the highest reconstruction accuracies are sampled on-axis. Additionally, as the receiver samples a larger area, it is able to roam a larger radius with a low K-BER.

DSDM mediates “compression noise”^42^, which refers to the reconstruction error that arises from using only a portion of the entire diffracted beam. As long as the receiver aperture is smaller than the diffracted beam, compression noise exists, regardless of whether there is additional noise or not. Compression noise decreases as the receiver size increases. For a small roaming radius *R*, the K-BER drops sharply, and this drop occurs for smaller *R* with larger receiver size. Compression noise decreases quickly when the receiver size is so large that the receiver aperture covers most of the high-intensity central area of the far-field beam. As expected, DSDM with smaller roaming areas and larger receivers have the best performance.

### Influence of the fractal order

DSDM performance is significantly improved when we increase the FO of the transmitted fractal beam. As the FO increases, the accuracy over the roaming area increases and smaller receiver sizes are possible. The far field beam exhibits smaller self-similar speckle features and information is encoded at higher spatial frequencies. Therefore, when the FO is large, the detector image produced from an arbitrary subsection of the roaming area closely resembles the transmitted data.

**Figure 5:**
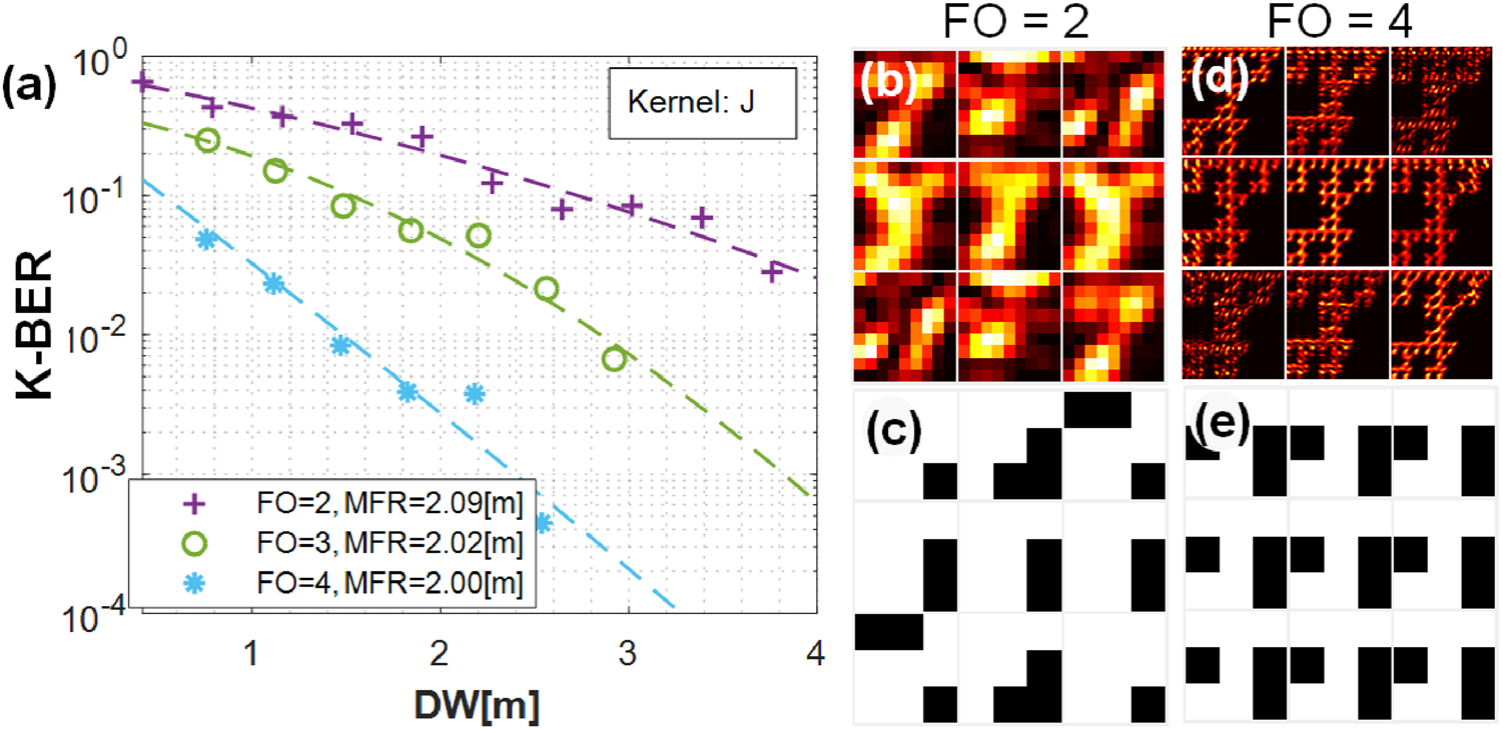
(**a**) K-BER trends with respect to fractal order FO = 2,3,4 as a function of receiver aperture size DW at $z = 10$ km. (**b**–**e**) The deconvolved images on an optical sensor and the reconstructed data for fractal orders FO = 2 (**b**, **c**) and FO = 4 (**d**, **e**) for different patches of the coverage area.

Figure 5a shows the K-BER versus DW at a propagation distance of *z* = 10 km and compares the K-BER performance of FO = 2, 3, and 4. The trend clearly shows that higher fractal orders achieve higher accuracy. The K-BER for FO = 4 is lower than the K-BER for FO = 2 and 3. In order to reach the same K-BER level of 10$^{-3}$, the FO = 3 channel needs a receiver size that is about 1.6 times larger than that of the FO = 4 channel. To put the receiver sizes into perspective, at Fig. 5a, when FO = 4, the K-BER of 10$^{-3}$ is achieved with a receiver size less than 25% of the maximum roaming area (the receiver area is DW$^2$ = 6.25 m$^{2}$; maximum roaming area is 25 m$^{2}$). The Fourier-plane detector images carry more self-similar, iterated features with FO = 4 (Fig. 5d,e) compared to FO = 2 (Fig. 5b,c). The greater degree of redundancy in these features leads to smaller K-BER with higher FO.

Result indicates that enlarging FO for transmitted fractals is an effective way to improve system performance of DSDM. However, larger FO beams require more transmitted pixels, which demand longer propagation distances for encoding (Eq. 5). Smaller pixels may be used to decrease the necessary propagation distance but this also increases the rate of beam divergence. Thus, we need to balance between the pixel size, propagation distance and computational window size.

### Influence of the receiver size and kernel shape

**Figure 6:**
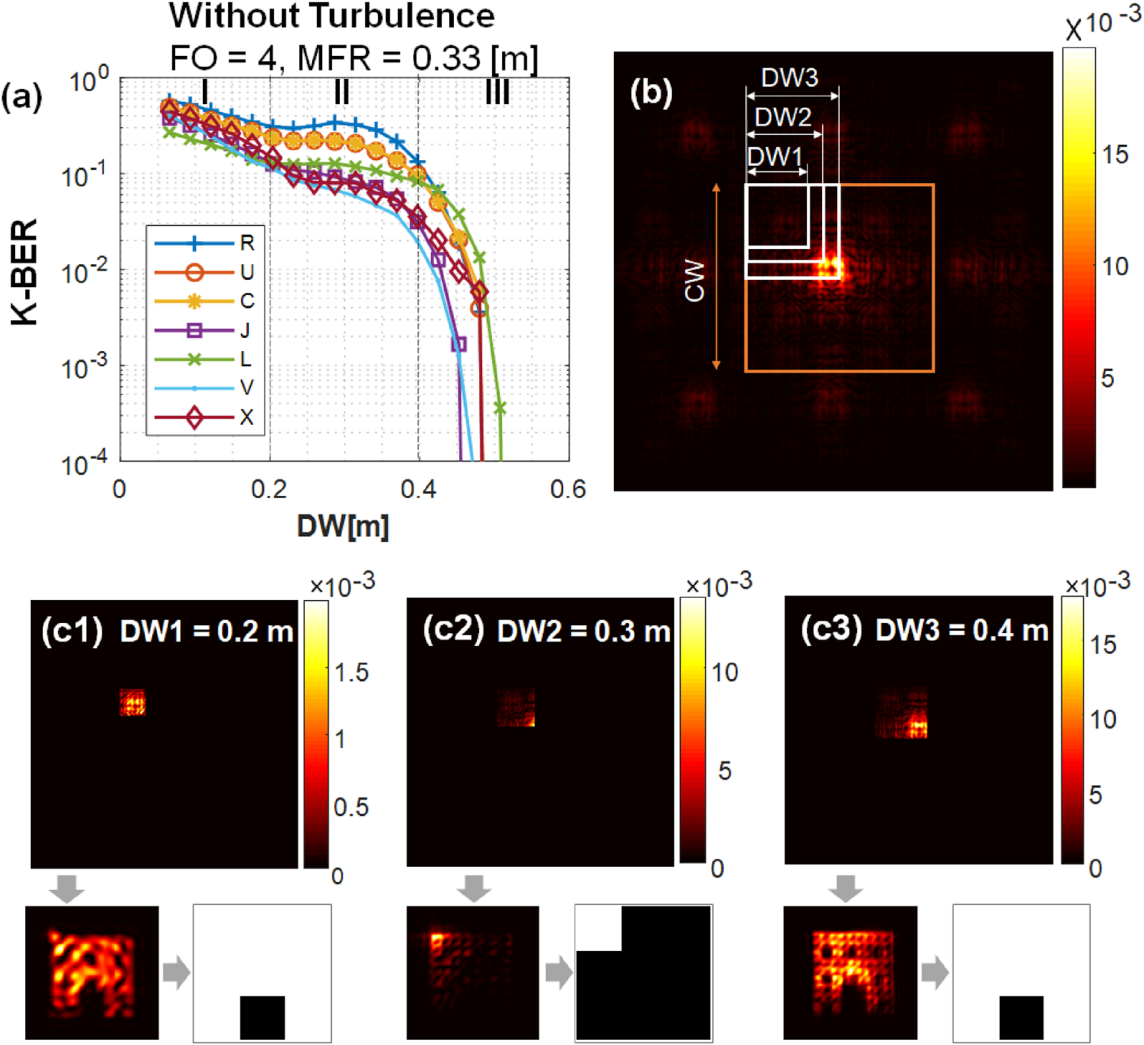
(**a**) K-BER versus receiver width (DW). The trend decreases with larger DW and depends on kernel data. There are three marked region (I, II, and III). The slightly rise in K-BER (marked zone II) is a result of minimal diffraction encoding or shorter propagation distance $z = 2.5$ km. (**b**) Far-field pattern for the kernel “R” showing the coverage width (CW) and 3 different receiver areas. (**c**) Corresponding detector patterns and reconstructed data for the receiver areas (**c1**) DW1 = 0.2 m, (**c2**) DW2 = 0.3 m, and (**c3**) DW3 = 0.4 m.

One advantage of DSDM is that the receiver aperture can be much smaller than the whole diffracted beam and capture the beam off-axis. This advantage varies with the propagation distance and kernel shape. In the previous graph’s trend for the kernel “J” where the K-BER vs DW relationship is smooth (Fig. 5a), the propagation distance $z =$10 km puts the receiver approximately in the far field or closer to $z_{DFF}$. At a shorter distance $z=2.5$ km, the K-BER versus DW for different kernels shows more subtle features (Fig. 6a] resulting from partial diffraction encoding. The inflection points in the curves in Fig. 6a result from partial diffraction encoding. Compared to the smooth curve in Fig. 5a, two obvious turning points in Fig. 6a separate the trend lines into three regions.

The K-BER performance in Region I is again limited by compression noise^42^. By comparing Fig. 6c1,c3, we see the influence of compression noise: a detector image with larger receiver, where DW = 0.4 m, contains more detailed information than with a smaller receiver, DW = 0.2 m. The upper left corner of the receivers of different size are located at the same place (see white squares in Fig. 6b). In Region III, the K-BER drops sharply as before with increasing receiver size. Results show that when DW > 0.45 m (where the receiver size is 30% of the maximum roaming area), K-BER is below the forward error correction limit^43^ of $10^{-3}$ . This indicates that the K-BER is reduced simply by increasing DW.

However, the trend lines in Region II in Fig. 6a are flattened or slightly raised, which appears to be in violation of the trend described above. In this Region, the K-BER performance is dominated by partial diffraction encoding. Region II disappears at a longer propagation distance of $z=$ 10 km, which is closer to $z_{DFF}$ or the “far field” (Fig. 5a). Figure 6b shows different receiver sizes DW = 0.2, 0.3, and 0.4 m in the roaming area. Accurate reconstruction is achieved when DW = 0.2 and 0.4 m (Fig. 6c1,c3). One important area of future work will be the reconstruction of beams with partial diffraction encoding such as those in Region II. An illustration is shown in Fig. 6c2 when a receiver DW = 0.3 m is located in the top left of the far field. In this case, the bottom right corner of receiver samples only a part of the high-intensity central area. This area remains localized and is as large as the original, transmitted beam. Since the intensity of the central portion is much higher than the other sampled parts, the upper-left corner of the deconvolved image is much brighter. With our on/off threshold reconstruction algorithm, only the brightest area is considered as ﻿‘1’, whereas the other dark areas are ﻿‘0’﻿s. This sampling, in combination with the current threshold algorithm, results in a higher error probability with DW = 0.3 m than DW = 0.2 m.

We note that, in many cases, the numerical reconstruction algorithm fails to identify the kernel pattern even though the detector images would easily be classified by human visual inspection. We have tried other reconstruction algorithms besides the intensity thresholding: intensity-differential thresholding and image boundaries, for instance. These algorithms do, in some cases, reduce the error probability compared to our simple intensity threshold approach. The simplest reconstruction algorithm, however, distills clearer understanding of the diffraction encoding. In the future, we anticipate that more advanced reconstruction algorithms will improve the accuracy beyond the results presented here.

## Discussion and conclusion

DSDM leverages the fact that fractal patterns of kernel data are redundantly encoded over large areas as they propagate to the far field. As a result, a small portion of the diffraction-encoded beam carries sufficient information to reproduce the original kernel data. Provided that the receiver detector has sufficient sensitivity, the best reconstruction accuracy is achieved from beams that have propagated to the far field. Although diffractals exhibit considerably more stable propagation in the far field, we are able to achieve the same K-BER with larger DW in the mid-field ($z<z_{DFF}$). We provide an analysis of the dependence on the receiver size and influence of fractal order in the mid-field.

Additionally, we have shown that diffractal beam divergence and propagation to the far field depend strongly on kernel shape. We have limited our study to the image reconstruction accuracy for a fixed kernel (K-BER). This limited scope is important since not all kernel patterns achieve the same K-BER. A fixed kernel pattern is useful alone as a spatial channel marker for FSO acquisition, tracking, and pointing^16^ and illustrates how DSDM would enable the alignment and channel locking between FSO transmitters and receivers. Random kernel patterns could be applied so that the channel aggregate capacity of DSDM would be the logarithm of the number of independent, transmitted kernel patterns. In other words, if all 512 combinations of $3\times 3$ kernels are transmitted, DSDM provides $\log _2 (512) = 9$ bits per frame.

In conclusion, we show enormous potential for DSDM in FSO communication and channel marking. Simulation results shows that with $81\times 81$ transmitted pixels, we achieve K-BER of 10$^{-3}$ when the receiver sizes are 30$\%$ of the maximum roaming area over propagation distances of 2.5 km. These results would be further improved with higher FO. DSDM may be also utilized in other applications where the alignment between transmitter and receiver is not fixed, either when a receiver “roaming area” is needed, or when an object needs to be marked or tracked. For example, FSO acquisition, pointing, and tracking.

In future work, the robustness of DSDM under atmospheric turbulence may be simulated and compared to other space-division multiplexing (OAM multiplexing for example) in FSO communication. Furthermore, the implementation of neural networks may improve the system accuracy. We have shown that, at times, the detector images are inaccurately classified using our linear threshold algorithm but easily classified by visual inspection. Judging from related research^44,45^, the incorporation of a neural network or optimization scheme could improve the K-BER by several orders over already-promising results.
